# Constrained evolution of the sex comb in *Drosophila simulans*

**DOI:** 10.1111/jeb.13015

**Published:** 2016-12-16

**Authors:** M. S. Maraqa, R. Griffin, M. D. Sharma, A. J. Wilson, J. Hunt, D. J. Hosken, C. M. House

**Affiliations:** ^1^ Centre for Ecology and ConservationCollege of Life & Environmental SciencesUniversity of ExeterCornwallUK; ^2^ Department of BiologyUniversity of TurkuTurun YliopistoFinland; ^3^ School of Science and HealthWestern Sydney UniversityPenrithNSWAustralia

**Keywords:** *Drosophila*, genetic constraints, post‐copulatory selection, precopulatory selection, selection gradients, sex combs

## Abstract

Male fitness is dependent on sexual traits that influence mate acquisition (precopulatory sexual selection) and paternity (post‐copulatory sexual selection), and although many studies have documented the form of selection in one or the other of these arenas, fewer have done it for both. Nonetheless, it appears that the dominant form of sexual selection is directional, although theoretically, populations should converge on peaks in the fitness surface, where selection is stabilizing. Many factors, however, can prevent populations from reaching adaptive peaks. Genetic constraints can be important if they prevent the development of highest fitness phenotypes, as can the direction of selection if it reverses across episodes of selection. In this study, we examine the evidence that these processes influence the evolution of the multivariate sex comb morphology of male *Drosophila simulans*. To do this, we conduct a quantitative genetic study together with a multivariate selection analysis to infer how the genetic architecture and selection interact. We find abundant genetic variance and covariance in elements of the sex comb. However, there was little evidence for directional selection in either arena. Significant nonlinear selection was detected prior to copulation when males were mated to nonvirgin females, and post‐copulation during sperm offence (again with males mated to nonvirgins). Thus, contrary to our predictions, the evolution of the *D. simulans* sex comb is limited neither by genetic constraints nor by antagonistic selection between pre‐ and post‐copulatory arenas, but nonlinear selection on the multivariate phenotype may prevent sex combs from evolving to reach some fitness maximizing optima.

## Introduction

Male sexually selected traits typically evolve rapidly (Andersson, 1994; Arnqvist 1998) through both pre‐ and post‐copulatory sexual selection (Partridge & Halliday, 1984). Precopulatory mechanisms of sexual selection include male–male competition and female mate choice, and post‐copulatory mechanisms of sexual selection include sperm competition and cryptic female choice (Parker, 1970; Eberhard, 1985; Andersson & Simmons, 2006; Hunt *et al*., 2009). Given the complexity of the mechanisms of sexual selection, an understanding of the form and strength of selection that pre‐ and post‐copulatory sexual selection impose is required to gain an understanding of the extravagance of the traits that they produce (Hunt *et al*., 2009).

In the last decade, an increasing number of studies have used multivariate statistical techniques to describe the form and strength of selection on sexually selected traits (reviewed in Hunt *et al*., 2009; Kingsolver & Diamond, 2011), and it is striking that directional selection is the dominant form of selection that has been documented (Hunt *et al*., 2009; Kingsolver & Diamond, 2011). This is intriguing as, theoretically, populations should evolve towards areas of high fitness on fitness landscapes (Phillips & Arnold, 1989; Kingsolver & Diamond, 2011), and as populations move closer to these regions, selection should become stabilizing with moves in any direction acting to lower fitness (Chenoweth *et al*., 2012). There are a number of mechanisms that may explain why populations never reach peaks on a fitness landscape, but one explanation is the presence of trade‐offs that could arise either from the genetic covariance structure among traits under selection or from antagonism of selection on the multivariate phenotype across episodes of selection (e.g. pre‐ and post‐copulatory episodes).

Genetic constraints may arise due to associations among traits (i.e. the genetic covariance structure), so selection on one will indirectly select on others (Cheverud, 1984; Phillips & Arnold, 1989; Blows & Brooks, 2003; Moore *et al*., 2004; Bentsen *et al*., 2006; Hunt *et al*., 2007a; Pitcher *et al*., 2014). If the genetic covariance or correlation (*r*_G_) between traits is negative with respect to each trait's (directional) effect on fitness (e.g. *r*_G_ < 0 between two positively selected traits), this should limit selection towards an adaptive peak (Fear & Price, 1998; Blows & Hoffmann, 2005). Evidence consistent with bivariate genetic constraints has been found in a cricket (*Gryllus lineaticeps*; Wagner *et al*., 2012), dung beetle (*Onthophagus taurus*; House & Simmons, 2005) and a cockroach (*Nauphoeta cinerea*; Moore *et al*., 2004). More recently, however, a focus on bivariate correlations to infer constraints has been criticized, as the data from long‐term studies suggest that populations do not evolve as predicted from bivariate genetic architecture alone (reviewed in Walsh & Blows, 2009). Instead, a multivariate approach that combines the genetic variance–covariance (**G**) matrix (i.e. the genetic variance across a suite of traits and the genetic covariances among them) with the vectors of linear selection gradients (***β***) (i.e. estimation of linear selection across suites of traits) has been advocated to assess the potential for genetic constraints (Walsh & Blows, 2009; Walling *et al*., 2014).

If trade‐offs occur between traits, they can also occur across discrete episodes of selection if trait values that increase fitness in one selective bout decrease it in another (Andersson & Simmons, 2006; Hunt *et al*., 2009; Kingsolver & Diamond, 2011). For instance, if selection on a trait is positive during mate acquisition and negative during sperm competition, this can result in no net selection on traits (Hunt *et al*., 2009). However, the empirical evidence for these sorts of trade‐offs is mixed. For example, pre‐ and post‐copulatory selection appear to be reinforcing in the guppy (*Poecilia reticulata*; Evans *et al*., 2003), cricket (*Acheta domesticus*; Head *et al*., 2006), fly (*Drosophila simulans*; Hosken *et al*., 2008) and stalk‐eyed fly (*Teleopsis dalmanni*; Rogers *et al*., 2008). In contrast, episodes of pre‐ and post‐copulatory selection are antagonistic in the water strider (*Gerris lacustris*; Danielsson, 2001), dung beetles (*Onthophagus* species; Simmons & Emlen, 2006), firefly (*Photinus greeni*; Demary & Lewis, 2007), gulf pipefish (*Syngnathus scovelli*; Rose *et al*., 2013) and the flour beetle (*Gnatocerus cornutus*; Okada *et al*., 2014). So at least sometimes, the trait values that would be of highest fitness in one selective episode may not be highest in another selective bout, and therefore, evolution is constrained by antagonistic selection.

Many male *Drosophila* have a secondary sexual trait on their forelegs, the sex comb(s) (Kopp & True, 2002). These are used to grasp the female's abdomen and genitalia prior to and during copulation. The design of the sex combs is highly variable across closely related species, with comb and tooth number being especially variable (Markow *et al*., 1996). Field and laboratory studies provide evidence that these interspecific patterns of phenotypic variation are partly due to sexual selection. For instance, during precopulatory sexual selection there is positive (directional) selection on comb size and comb symmetry in *D. bipectinata* (wild population; Polak *et al*., 2004), whereas positive selection on tooth number has been reported in *D. melanogaster* (experimental lines; Promislow *et al*., 1998). There is also post‐copulatory selection on sex comb traits in *D. bipectinata*, with positive selection on comb size (artificial lines; Polak & Simmons, 2009) and nonlinear (disruptive) selection against intermediate tooth number in *D. melanogaster* (wild populations; Robinson *et al*., 2012). However, a number of other studies have found less evidence for selection. For instance, no relationship between sex comb tooth number and mating success was found in either *D. melanogaster* (wild populations; Markow *et al*., 1996; experimental lines; Snook *et al*., 2013) or *D. pseudoobscura* (experimental lines; Snook *et al*., 2013). This poses a paradox because although *Drosophila* sex combs have characteristics expected of a sexually selected trait (e.g. rapid divergence among lineages), the evidence that these characters are under strong sexual selection is inconsistent. One resolution may be that sex comb traits are the target of selection that has not been measured and/or selection on sex combs across pre‐ and post‐copulatory selection is antagonistic.

In this study, we investigate the hypothesis that the evolution of the paired *D. simulans* sex comb is constrained by genetic constraints and/or antagonistic selection across episodes of sexual selection. Sexual selection has been intensely studied in *D. simulans* for a number of traits (e.g. Hosken *et al*., 2008; Taylor *et al*., 2008a,b; Ingleby *et al*., 2014), and previous research suggests that sex comb tooth number is under negative directional selection through precopulatory mating success (Markow *et al*., 1996). However, tooth number represents just one component of the multivariate comb phenotype and little is known about whether (and how) selection differs depending on whether it occurs pre‐ vs. post‐copulation. It is also unknown whether precopulatory selection is itself contingent on whether females have previously mated. Nonetheless, prior work has shown the single sex comb on the fore‐tarsus of this species is functionally important, being used to grasp the female abdomen and genitalia and spread her wings prior to and during copulation (Sharma *et al*., 2011). We therefore expect that overall comb morphology will be subject to directional selection. To start, we used a half‐sib breeding design to estimate the genetic variance for and covariances among components of the sex comb (and body size). Next, we quantified the form and strength of sexual selection across four episodes of sexual selection: precopulatory selection when females were virgin or mated and post‐copulatory sexual selection during sperm competition, when the focal male was first to mate (i.e. P1, sperm defence) or second to mate (i.e. P2, sperm offence).

## Materials and methods

### Fly stocks

Our laboratory wild‐type populations of *D. simulans* were derived from 20 isolines (supplied by Centre for Environmental Stress and Adaptation Research, La Trobe University, Australia) that originally came from individuals that were caught in Tuncurry, eastern Australia, in March 2004. In the laboratory, these isolines were mixed and maintained for at least 7 years prior to the start of this study and have been found to be genetically and phenotypically variable for all traits that have been assayed (Hosken *et al*., 2008; Wright *et al*., 2009; Okada *et al*., 2011; Sharma *et al*., 2011). In addition to the wild‐type population, laboratory populations of ebony flies, which carry a homozygous recessive phenotypic marker, were derived from a strain obtained from the Tucson stock centre and maintained as above for over 50 generations. The grey‐black cuticle of ebony flies allows the easy discrimination between progeny of ebony females sired by ebony vs. wild‐type males (Ashburner *et al*., 2005). All population cages (wild‐type and ebony) had an excess of 600 flies with overlapping generations and free mate choice. All stock and experimental offspring were maintained at 25 °C under a 12 : 12‐h light:dark cycle and maintained on *Drosophila* culture medium (Jazz Mix Drosophila Food, Fisher Scientific; and Drosophila Quick Mix Medium, Blades Biological) with an excess of food. This reduces the risk of environmental influences affecting mating and remating probabilities because of stress response (Zera & Harshman, 2001).

### Breeding design

#### Parental generation

For our experimental breeding design, wild‐type flies were initially collected from population cages. Egg laying vials were placed in the cages of two wild‐type populations daily and left for 24 h. These vials were incubated until peak eclosion (ca. 8–9 days after egg laying). Offspring that eclosed overnight were killed and virgins were collected ca. 7 h later (Sharma *et al*., 2010). Virgin males were maintained in standard culture vials, with ca. 80 males per vial. Virgin females were aspirated into ca. 800 individual vials containing culture medium. These virgin females and males were the parents for our design and were 3 days old before breeding commenced to ensure full sexual receptivity (Manning, 1967).

#### Breeding and rearing

A conventional half‐sibling breeding design was used (Lynch & Walsh, 1998), where 130 sires were each mated with five dams. Details of the mating regime are as follows: a sire was housed with a randomly selected, virgin female for 24 h to maximize the probability that the pair would mate. The following day, the male was aspirated from the vial and transferred to a new vial that contained a virgin female for 24 h. The process was repeated three more times until the sire had been housed with a total of five dams. The mated dams were housed singly in oviposition vials and transferred daily to new oviposition vials for a total of 4 days. The oviposition vials were stored at 25 °C for 12 days under a 12/12‐h light:dark cycle until the offspring began to emerge. Six days after the first eclosion, the offspring were collected, labelled and frozen at −20 °C for subsequent dissection, measurement and quantitative genetic analysis.

### Multivariate sexual selection

#### Experimental design

For experimental mating assays, a sample of ebony and wild‐type flies (not the same as those that were used for the breeding design) were collected as virgins from population cages using the protocols described above (‘Parental generation *’*). Virgin females and males were used for mating trials when the females were 3 days old and males were 3–4 days old, to ensure full sexual receptivity (Manning, 1967). Mating trials began at the beginning of the photophase of the light:dark cycle as this is when the flies are most reproductively active (Sakai & Ishida, 2001). In all trials, each male was aspirated into a female housing vial and continuously observed for 2 h during which courtship (i.e. wing flicking, wing vibration, leg rubbing and licking) and mating were recorded (Spieth, 1974).

#### Sex comb morphology and precopulatory sexual selection

In the first part of the study, we investigated whether variation in sex comb morphology predicts mating success with virgin females (Virgin Trial) or with mated females (Nonvirgin Trial). To do this, we used no‐choice mating assays that are a standard method to assess overall male attractiveness (e.g. Hedge & Krishna, 1997; Koref‐Santibanez, 2001; Gowaty *et al*., 2002; Yenisetti & Hedge, 2003; Shackleton *et al*., 2005), and the results of assays with single males and multimales are the same (Taylor *et al*., 2008a,b). During Virgin Trials, males that courted but were rejected (*n* = 154) or courted and mated (*n* = 340, total *n* = 494) were separated from the females and frozen at −20 °C for morphometric measurement. During Nonvirgin Trials, we used a new set of flies that were derived from the same stock population. The females were once mated, but detailed observation of their mating behaviour was not recorded. All females were 7 days old, having mated 4 days before their second exposure to virgin males. The mating procedure in this trial was identical to that described above (Virgin Trial). All males that courted but were rejected (*n* = 329) or courted and mated (*n* = 154, total *n* = 483) were frozen at −20 °C for morphometric measurement.

#### Sex comb morphology and post‐copulatory sexual selection

In the second part of the selection study, we investigated whether variation in sex comb morphology predicts fertilization success. Ebony females were sequentially mated with a focal, wild‐type male followed by an ebony male (paternity defence – P1) or an ebony male followed by a focal, wild‐type male (paternity offence – P2). Males mated once only and in a single role – defensive or offensive. During the observation period, if copulation occurred, the male was removed from the chamber, aspirated into an Eppendorf tube and stored at −20 °C for dissection and measurement. Following the first mating, females were transferred daily into fresh food vials to oviposit for 4 days before their second exposure to virgin males. The second mating procedure for mated females was identical to that described above. Ebony females that did not mate with the second mating partner during the 2‐h assay were excluded from the data set, along with their first mate (*n* ~ 600 – *D. simulans* are reluctant to mate, particularly with mutant strains). Following their second mating, twice‐mated females were once again transferred daily into fresh food vials to oviposit for 4 days. On the 5th day, the female was aspirated into an Eppendorf tube and stored at −20 °C. Vials that had contained the mated females were stored at 25 °C and monitored daily until offspring emerged. Seven days after the first emergence, the vials were inverted and stored in the freezer and the ebony and wild‐type offspring from each of the female's eight vials was subsequently counted to determine the number of offspring that were sired by the focal (i.e. wild‐type) male during defensive (P1, *n* = 308) or offensive mating (P2, *n* = 355).

#### Dissection and morphometric measurement

**Figure 1:**
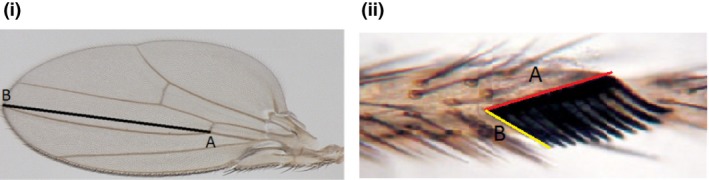
Morphological measures of male *Drosophila simulans*: (i) wing and (ii) sex comb. The length of the wing was measured as the distance between points a and b. Three components of the sex comb were measured: comb length (CL; a); tooth length (TL), which was measured as the average length of the first (b), third and fifth teeth; and comb tooth number (TN).

The left and right forelegs and wings of focal, wild‐type males or sons from our breeding design were carefully pulled free from the body of each male and then mounted on glass slides in a droplet of Hoyer's medium. Digital images for wings (×30) and sex combs (×100) were captured using a Leica dissecting microscope (M125) connected to a Leica camera (DFC295). Wing length and sex comb components were measured using ImageJ v1.46r (RSB National institute of Mental Health, USA) (Fig. 1).

We used wing length (WL) as an index of body size (Markow & Ricker, 1992; Gilchrist & Partridge, 1999; Sharma *et al*., 2011), and both left and right wings of each male were measured and an average value was calculated. Three components of sex comb morphology were measured: the comb length (CL); tooth length (TL), measured as the average length of the first, third and fifth teeth); and comb tooth number (TN) (Fig. 1). All sex comb characteristics including CL, TL and TN were estimated as the average of the measurements on the left and right body sides. The precision of the measurements was assessed by blindly measuring all traits twice on a subsample of wings and sex combs (*N* = 20). Two measures of the same trait were tightly correlated (TL: *r* ^2^ = 0.919, *P* < 0.05; CL: *r* ^2^ = 0.982, *P* < 0.001; TN: *r* ^2^ = 1.00, *P* < 0.001; WL: *r* ^2^ = 0.992, *P* < 0.001).

### Statistical analysis

#### Genetic analyses

Data were analysed using animal models fitted with restricted maximum likelihood in ASReml (version 3.0; VSN International Ltd) with assumed Gaussian errors (see Wilson *et al*., 2010). First, we tested for additive genetic variance using univariate models fitted to each of the sex comb component traits (comb length CL, tooth length TL and tooth number TN) and size (wing length WL). Each model contained the mean as a fixed effect and random effects of additive genetic merit and a ‘maternal identity’ effect. The latter was included to protect against upward bias from maternal (or other common environment) effects shared by full‐sibs. For each trait, we compared this to a reduced model with the additive effect dropped using a likelihood ratio test and assuming that twice the difference in log‐likelihoods is distributed as a 50 : 50 mix of $\chi_{1}^{2}$ and $\chi_{0}^{2}$ (subsequently denoted $\chi_{0,1}^{2}$). Having detected significant genetic variance in all traits (see Results), we formulated a multivariate animal model which was used to estimate the additive variance–covariance matrix (**G**) and derived parameters. To facilitate convergence in the multivariate model, traits were scaled to unit variance by dividing by their (observed) standard deviations. Heritability (*h* ^2^) was estimated for each trait as *V*_A_/*V*_P_, where *V*_A_ is the additive genetic variance and *V*_P_ is the phenotypic variance, determined as the sum of *V*_A_, *V*_M_ (maternal variance) and *V*_R_ (residual variance). We similarly estimated the magnitude of the maternal effect as *m* ^2^, where *m* ^2^ = *V*_M_/*V*_P_. Genetic correlations (*r*_G_) were determined for each pair of traits (1,2) as *r*_G(1,2)_ = COV_A(1,2)_/(*V*_A1_*V_A2_)^0.5^, where COV_A_ is the estimated additive genetic covariance. For comparison, we also estimated the corresponding phenotypic correlations *r*_P_.

#### Multivariate selection analysis

To determine whether male phenotypic traits (CL, TL, TN and WL) influenced fitness during precopulatory or post‐copulatory selection, we used a standard multivariate selection analysis approach. In precopulatory bouts of selections, a male was assigned a score of 1 if the male courted and mated and a 0 if the male courted only. In these mating success trials, the female was always presented with a wild‐type male to increase the likelihood that a male would attempt to court and mate. As a consequence, we would have been unable to determine the number of offspring that were sired by the focal male when mating a previously mated female without extensive genotyping work, hence the binary fitness measure. In post‐copulatory, fertilization success trials, male fitness was assigned a continuous value – the number of offspring that were sired by the focal male which ranged from 0 to 200. The mating and fertilization success response variables were transformed to relative fitness by dividing individual scores by the mean for each data set. The male phenotypic traits were standardized to zero means and unit variances as suggested by Lande & Arnold (1983). We then fitted a separate linear multiple regression for each of the four bouts of selection to estimate linear selection gradients when females were virgins (*β*_v_) or previously mated (*β*_m_) or the focal male mated in a defensive role (*β*_P1_) or an offensive role (*β*_P2_) (Lande & Arnold, 1983). Next, we applied a quadratic regression model including all linear, quadratic and cross‐product (i.e. correlational) terms to estimate the matrix of nonlinear selection gradients for males when females were virgin (*γ*_v_) or previously mated (*γ*_m_) or the focal male mated in a defensive role (*γ*_P1_) or an offensive role (*γ*_P2_). Quadratic regression coefficients were doubled to yield the standardized nonlinear selection gradients (see Stinchcombe *et al*., 2008). As our binary and continuous fitness measures did not conform to a normal distribution, we used a resampling procedure to assess the significance of our linear and nonlinear selection gradients. Our fitness scores were randomly shuffled across individual phenotypes 10 000 times to generate a null distribution of pseudoselection gradients expected in the absence of a causal phenotype–fitness relationship (Mitchell‐Olds & Shaw, 1987). The probability that the gradient pseudo‐estimate was equal to or less than the original estimated gradient (out of 9999 permutations) was then tested. We conducted separate randomization analyses for the multiple regression models for directional selection (i.e. model containing only linear terms) and for the full quadratic model (i.e. model containing linear, quadratic and correlational terms).

To establish the extent of nonlinear selection acting on male phenotypic traits, we conducted a canonical analysis using the approach suggested by Reynolds *et al*. (2010). The analysis generates a new matrix that consists of vectors of linear selection described by theta (***θ***_*i*_) and nonlinear selection that are described by eigenvalues (**λ**_*i*_) and their corresponding eigenvectors (**m**_*i*_). Tests of the significance of the eigenvalues were conducted using the permutation procedure outlined in Reynolds *et al*. (2010). We used thin‐plate splines (Green & Silverman, 1994) to visualize the major axes of the fitness surfaces extracted from the canonical rotation of **γ**_m,_ and **γ**_P2_. Tps functions in the fields package of R (version 2.13.0; available via http://www.r-project.org) were used to fit spline surfaces using the value of the smoothing parameter (**λ**) that minimized the generalized cross‐validation (GCV) score. We then plotted surfaces in R using both the perspective and contour map views. Finally, to test whether the linear, quadratic and correlational selection gradients differed when females had previously mated compared to when males mated in the offensive role, we used a sequential model building approach (partial F‐test) (Draper & John, 1988; see Chenoweth & Blows (2005) for a detailed description of this procedure).

## Results

### Genetic architecture

Comparison of full and reduced univariate models indicated significant additive genetic variance for comb length (CL: $\chi_{0,1}^{2}$ = 25.0, *P* < 0.001), tooth length (TL: $\chi_{0,1}^{2}$ = 5.48, *P* = 0.010), tooth number (TN: $\chi_{0,1}^{2}$ = 40.2, *P* < 0.001) and wing length (WL: $\chi_{0,1}^{2}$ = 4.78, *P* = 0.014). Estimates of maternal variance were nonzero in all cases except for TN where *V*_M_ was bound at zero (full results not shown), so we formulated the multivariate model with a 4 × 4 **G** matrix but a 3 × 3 maternal effect covariance matrix (i.e. no maternal effect on TN). Under this multivariate model, *h* ^2^ estimates for sex comb components ranged from moderate to high (Table 1). The heritability of wing length (which is a proxy for body size) was similar to previously published heritability of body size for *Drosophila* (*h* ^2^ ~ 0.4; Robertson, 1957; ~0.5; Coyne & Beecham, 1987). All genetic correlations between sex comb component traits were positive and nominally significant (based on |*r*_G_| > 2SEs; Table 2). Genetic correlations between wing length and all sex comb components were also positive although not significantly for WL and TL. While noting that estimated standard errors are approximate and so not necessarily robust for formal inference, the model was a significantly better fit to the data than a reduced version in which all off‐diagonal (i.e. COV_A_) terms in the **G** matrix were constrained to zero ($\chi_{6}^{2}$ = 112, *P* < 0.001). Thus, it is clear that **G** contains significant additive genetic covariance among the traits, and estimates are uniformly positive across all trait pairs.

**Table 1:** Phenotypic means and estimates of heritability (*h* ^2^) and maternal effect (*m* ^2^) for male body size and sex comb components (*N* sires = 110, *N* offspring = 1449). Estimates are from the multivariate animal model (see text for details)

Trait (unit)	Mean	*h* ^2^ (SE)	*m* ^2^ (SE)
Comb length (μm)	58.23 ± 0.12	0.61 ± 0.08	0.07 ± 0.02
Tooth length (μm)	39.23 ± 0.05	0.26 ± 0.11	0.27 ± 0.05
Tooth number	9.90 ± 0.02	0.53 ± 0.06	NA
Wing length (mm)	1154.08 ± 1.12	0.45 ± 0.06	0.40 ± 0.06

**Table 2:** Additive genetic correlations above the diagonal and phenotypic correlations below the diagonal for sex comb components: comb length (CL), tooth length (TL), tooth number (TN) and wing length (WL). Significant genetic (|*r*_G_| > 2SEs) and phenotypic correlations are in bold (after Bonferroni correction)

	CL	TL	TN	WL
Comb length (CL)		**0.31 ± 0.15**	**0.89 ± 0.03**	**0.64 ± 0.10**
Tooth length (TL)	**0.27 **±** **0.02		**0.89 ± 0.03**	0.27 ± 0.23
Tooth number (TN)	**0.84 **±** **0.01	0.01 ± 0.03		**0.70 ± 0.11**
Wing length (WL)	**0.45 **±** **0.02	**0.44 **±** **0.02	**0.23 **±** **0.02	

### Sexual selection on sex combs

Rather surprisingly, given the evidence from a previous study in *D. simulans* (Markow *et al*., 1996) we found no evidence of significant directional selection (i.e. ***β*** – linear selection that increases/decreases the trait mean) acting on any component of the sex comb in any of the four selective contexts (Table 3). However, we found evidence for nonlinear selection, which acted differently in each context. There are three different forms of nonlinear selection (i.e. **γ** coefficients that describe the curvature of nonlinear selection on individual traits): (a) stabilizing where **γ** coefficients are negative and individuals with intermediate trait values have highest fitness, (b) disruptive where **γ** coefficients are positive and individuals with extreme low or high trait values have highest fitness and (c) correlational selection where pairs of traits are jointly acted upon (Hunt *et al*., 2009). We find evidence for all three forms of nonlinear selection.

**Table 3:** The vector of standardized linear selection gradients (***β***) and the matrix of standardized nonlinear gradients (**γ**†) for sex comb morphological traits in male *Drosophila simulans* during precopulatory sexual selection when a male courted and/or mated a (A) virgin female or (B) nonvirgin female and during post‐copulatory selection in a competitive role when a male mated in a (C) defensive role (i.e. P1) or (D) offensive role (i.e. P2)

	***β***	**γ**			
		CL	TL	TN	WL
A. Standardized selection gradients when a male courted and/or mated a virgin female					
CL	0.020	0.264			
TL	0.019	−0.051	−0.012		
TN	−0.029	−0.161	−0.041	−0.012	
WL	−0.004	−0.108	0.027	0.196*	−0.004
B. Standardized selection gradients when a male courted and/or mated a nonvirgin female					
CL	−0.172	0.104			
TL	−0.051	0.031	−0.300*		
TN	0.010	−0.249	−0.022	0.422*	
WL	0.121	0.069	0.228**	−0.017	0.270
C. Standardized selection gradients when a male mated in a defensive role (P1)					
CL	−0.108	−0.030			
TL	−0.010	0.099	−0.106		
TN	0.127	−0.118	0.041	0.228	
WL	−0.119	−0.018	−0.073	−0.121	0.234*
D. Standardized selection gradients when a male mated in an offensive role (P2)					
CL	−0.055	0.806**			
TL	0.038	0.025	−0.154		
TN	0.094	−0.509*	−0.026	0.25	
WL	−0.047	−0.282*	0.119	0.157	0.13

CL, comb length; TL, tooth length; TN, tooth number; WL, wing length. Randomization tests: *P <* *0.05, **P <* *0.01, ***P <* *0.001.

†Nonlinear selection gradients include quadratic ($z_{ii}^{2}$) gradients on the diagonal and correlational (*z*_*i*_ *z*_*j*_) gradients below the diagonal.

### Precopulatory sexual selection

Nonlinear selection was weak and nonsignificant when males courted virgin females with the exception of significant positive correlational selection between tooth number (TN) and wing length (WL) (Table 3A). Canonical rotation of the **γ** matrix of nonlinear selection gradients produced one positive and three negative eigenvalues, which describe the curvature of selection on the major axes of selection, rather than on individual traits (Table 4A; i.e. positive eigenvalue is indicative of disruptive selection along **m**_**1**_ and negative eigenvalue is indicative of stabilizing selection along **m**_**2**_ **–m**_**4**_). However, selection on the eigenvectors (**m**_**1**_–**m**_**4**_) was nonsignificant (Table 4A).

**Table 4:** Linear (***θ***_*i*_) and nonlinear (***λ***_*i*_, the eigenvalue) selection gradients and the M matrix† of eigenvectors (*m*_*i*_) from the canonical analysis of **γ** for (A) virgin mating success, (B) nonvirgin mating success, (C) P1 experiment and (D) P2 experiment

	***θ***_*i*_	***λ***_*i*_	M			
			CL	TL	TN	WL
A. Canonical analysis of virgin mating success						
**m**_**1**_	0.029	0.286	0.696	0.428	−0.575	−0.038
**m**_**2**_	0.015	−0.010	0.629	−0.148	0.623	0.441
**m**_**3**_	−0.020	−0.139	−0.021	−0.629	−0.530	0.568
**m**_**4**_	−0.011	−0.249	0.345	−0.632	−0.007	−0.693
B. Canonical analysis of nonvirgin mating success						
**m**_**1**_	0.086	0.565	**−0.485**	−0.059	**0.869**	−0.074
**m**_**2**_	0.076	0.004	−0.674	−0.379	−0.440	−0.456
**m**_**3**_	−0.132	−0.099	0.555	−0.573	0.223	−0.560
**m**_**4**_	−0.128	−0.515**	0.047	**0.724**	0.016	**−0.687**
C. Canonical analysis of P1						
**m**_**1**_	0.184	0.374	−0.145	0.133	0.715	−0.671
**m**_**2**_	0.049	0.173	−0.489	−0.258	0.542	0.633
**m**_**3**_	−0.070	−0.022	−0.614	−0.575	−0.379	−0.385
**m**_**4**_	−0.023	−0.200	0.602	−0.764	0.227	−0.041
D. Canonical analysis of P2						
**m**_**1**_	0.078	1.204*	**−0.824**	0.0003	**0.487**	0.288
**m**_**2**_	0.052	0.096*	−0.182	**−0.444**	0.197	**−0.855**
**m**_**3**_	0.047	−0.052	0.527	0.008	0.846	0.078
**m**_**4**_	−0.068	−0.216	0.095	−0.896	−0.090	0.424

Randomization tests: *P <* *0.05, **P <* *0.01, ***P <* *0.001.

†Values in bold, contributed most to that eigenvector (*m*_*i*_).

**Figure 2:**
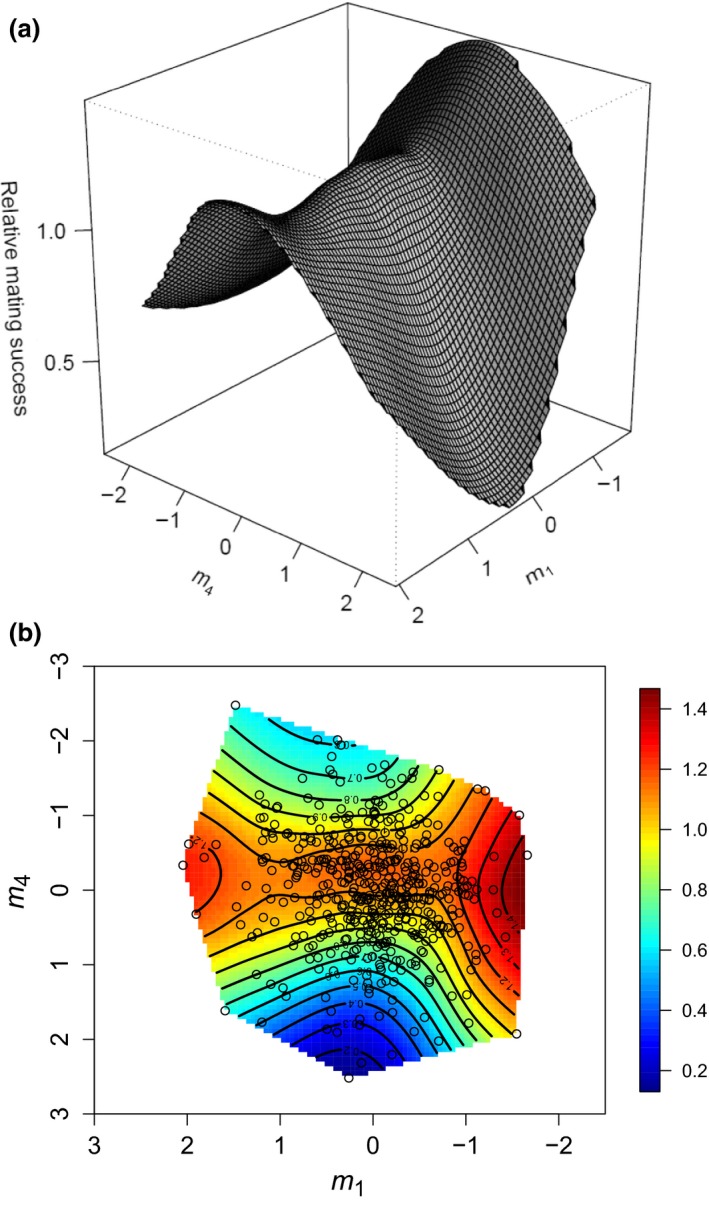
Thin‐plate spline visualizations (a: perspective view; and b: contour view) of the two major axes of nonlinear selection (**m**_**1**_ and **m**_**4**_) on the fitness surface when males courted nonvirgin females. In the contour view, red‐to‐orange coloration represents regions of highest fitness, whereas blue coloration represents regions of lowest fitness. Individual data points are provided as black circles on the surface.

Nonlinear selection was stronger when males courted nonvirgin females. There was significant stabilizing (negative **γ**) selection on tooth length (TL) and disruptive (positive γ) selection on the tooth number (TN) as well as positive correlational selection between tooth length (TL) and wing length (WL) (Table 3B). Canonical rotation of the γ matrix of nonlinear selection gradients produced a combination of disruptive selection along the **m**_**1**_ and **m**_**2**_ axis and stabilizing selection along the **m**_**3**_ and **m**_**4**_ axis; however, there was only significant selection along eigenvector **m**_**4**_ (Table 4B). This axis of significant selection for the nonvirgin mating phase shows stabilizing (negative **γ**) selection which we visualized with **m**_**1**_ that had the largest, albeit nonsignificant disruptive (positive γ) eigenvalue. These represent parts of the fitness surface that curve downwards and upwards respectively to create a saddle‐like fitness surface in the **m**_**1**_ **–m**_**4**_ plot (Fig. 2a). Along the **m**_**4**_ axis, highest fitness occurred along a ridge which corresponds with intermediate values and was heavily influenced by tooth length (TL) and wing length (WL) (i.e. in each row of M in Table 4, the magnitude of the values indicates the contribution of individual traits to an eigenvector). A contour‐view visualization of the same fitness surface with an overlay of the data points shows that many of the males are spread along the ridge on the **m**_**4**_ axis (Fig. 2b).

### Post‐copulatory sexual selection

Nonlinear selection was weak and nonsignificant when males mated in a defensive role with the exception of significant disruptive selection (positive **γ**) on wing length (Table 3C). Canonical rotation of the **γ** matrix of quadratic selection gradients produced a combination of disruptive selection along the **m**_**1**_ and **m**_**2**_ axis and stabilizing selection along the **m**_**3**_ and **m**_**4**_ axis; however, selection along these vectors (**m**_**1**_–**m**_**4**_) was nonsignificant (Table 4C).

**Figure 3:**
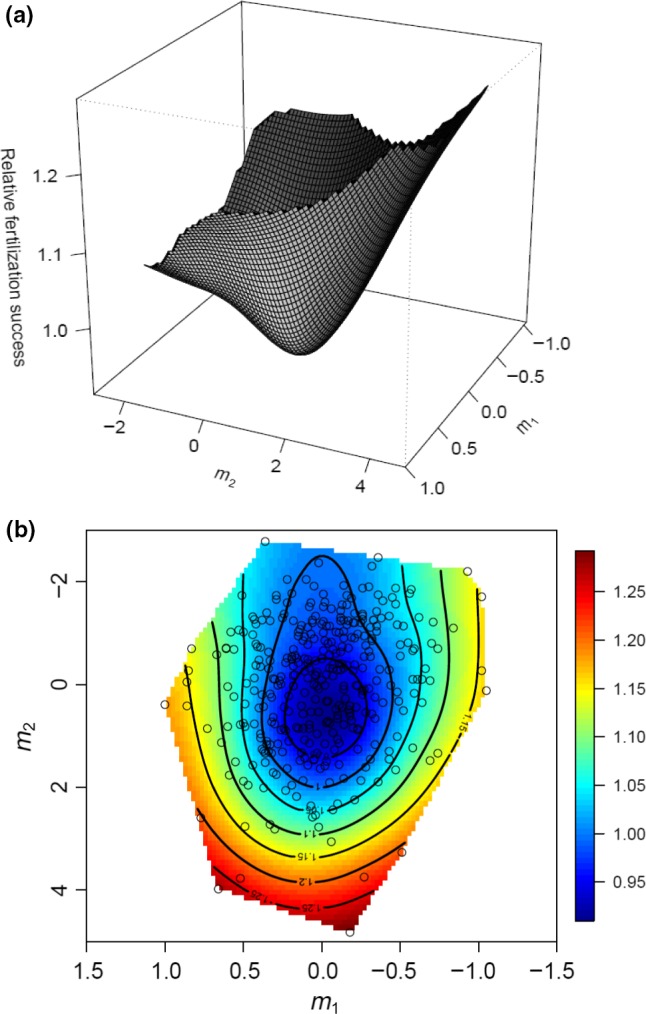
Thin‐plate spline visualizations (a: perspective view; and b: contour view) of the two major axes of nonlinear selection (**m**_**1**_ and **m**_**2**_) on the fitness surface when males mated in the offensive role (P2). In the contour view, red‐to‐orange coloration represents regions of highest fitness, whereas blue coloration represents regions of lowest fitness. Individual data points are provided as black circles on the surface.

Nonlinear selection was stronger during competitive mating when males mated in the offensive role (P2). There was disruptive (positive **γ**) selection on comb length (CL) and negative correlational selection between comb length (CL) and tooth number (TN) and comb length (CL) and wing length (WL) (Table 3D). Canonical rotation of the γ matrix of quadratic selection gradients produced a combination of disruptive selection along the **m**_**1**_ and **m**_**2**_ axis and stabilizing selection along the **m**_**3**_ and **m**_**4**_ axis, but selection along these vectors was only significant for **m**_**1**_ and **m**_**2**_. These axes of significant selection for the competitive, offensive mating phase (P2) showed disruptive selection along the **m**_**1**_ and **m**_**2**_ axes which curved the fitness upwards to create an inverted fitness surface in the **m**_**1**_–**m**_**2**_ plot (Fig. 3a). Along the ridge of highest fitness (i.e. intermediate values of **m**_**1**_ and positive values of **m**_**2**_), high paternity was correlated with a long sex comb, few but long comb teeth and large body size. However, a contour‐view visualization of the same fitness surface with an overlay of the data points shows that few males occupy this region on the landscape (Fig. 3b).

### The strength and form of linear and nonlinear selection across episodes

To test for possible differences in selection on the sex comb and body size (i.e. WL) during bouts of significant pre‐ and post‐copulatory sexual selection, we compared the strength of linear, quadratic and correlational selection across selective bouts. The strength of linear (*F*_4,826_ = 1.192, *P *=* *0.313), quadratic (*F*_4,818_ = 1.576, *P *=* *0.179) and correlational selection (*F*_6,806_ = 0.469, *P *=* *0.759) did not differ significantly between these bouts of selection.

## Discussion

We find that there is substantial genetic variation in the male sex comb trait components which are positively genetically correlated with each other and with body size. However, there was no evidence of directional selection on the sex comb across any bout of sexual selection. Thus, contrary to our predictions, it is the absence of directional selection that is the primary limitation to the evolution of the *D. simulans* sex comb rather than genetic constraints arising from among‐trait covariance and/or antagonistic linear selection across episodes of selection. Whilst evidence for linear selection was conspicuous by its absence, we did find complex patterns of significant nonlinear selection. In particular, we found disruptive selection acting on male sex combs during post‐copulatory selection when females are already mated.

### Genetic (co)variance among components of the sex comb

A breadth of studies find that sexually selected traits harbour abundant genetic variation (reviewed in Roff & Mousseau, 1987; Houle, 1992; Pomiankowski & Moller, 1995; Walsh & Blows, 2009). Our average *h* ^2^ estimate for sex comb components (*h* ^2^ = 0.46) is high and comparable with other *h* ^2^ estimates for morphological traits (Roff & Mousseau, 1987; Houle, 1992; Pomiankowski & Moller, 1995). The maintenance of genetic variation in sexually selected traits is an evolutionary puzzle, and a number of models have been developed to explain the phenomena (Taylor & Williams, 1982; Mousseau & Roff, 1987; Pomiankowski & Moller, 1995; Rowe & Houle, 1996). Here, it appears that the lack of significant directional selection coupled with stabilizing and disruptive selection (which may promote genetic variation) has maintained genetic variance in the sex comb. We also found positive genetic correlations between component traits of the comb and body size which should result in positively correlated indirect selection responses.

### Linear selection on the sex comb across selective episodes

A previous study of *D. simulans* found that directional selection during precopulatory sexual selection favoured fewer teeth in the comb, whereas we found no evidence that directional selection acts on sex comb components during any bout of selection. More generally, the evidence that selection acts on components of the sex comb of *Drosophila* species is mixed. In part, this may be a result of experimental design – typically, estimates of selection on the sex comb are univariate (Markow *et al*., 1996; Promislow *et al*., 1998; Polak *et al*., 2004; Polak & Simmons, 2009; Snook *et al*., 2013) even though this may underestimate the strength of selection (Blows & Brooks, 2003). For instance, if nonlinear selection was acting, it could result in linear selection gradients being estimated that simply cross two points of a nonlinear selection gradient (Hunt *et al*., 2009). The results of this study, and from a field study of *D. melanogaster*, where sexual selection on the sex comb was disruptive (Robinson *et al*., 2012), suggest that this may be an oversight as nonlinear selection was the dominant form of selection.

Given that directional selection on male sex comb components was absent, it is clear that trade‐offs (between component traits and/or pre‐ vs. post‐selective episodes) are neither present nor required to explain evolutionary stasis. Among previous studies of sexual selection on male traits, precopulatory selection for elaborate male traits is often reinforced by post‐copulatory fertility benefits (Rogers *et al*., 2008), sons with high fertilization success (Hosken *et al*., 2008) and/or high‐quality sons (Head *et al*., 2006). In *D. simulans*, precopulatory selection acting on the sex comb is weak, so it seems unlikely that females exercise mate choice on the basis of male sex comb morphology. Furthermore, during post‐copulatory sexual selection more than one sex comb phenotype is correlated with fertilization success during competitive mating, and therefore, it seems unlikely that sex combs provide a clear signal of sire or offspring reproductive quality.

### Nonlinear selection on the sex comb across selective episodes

When females were already mated, precopulatory sexual selection favours males with intermediate tooth length (TL) and wing sizes which resulted from a blend of stabilizing and correlational selection on these traits. A similar pattern of stabilizing selection has been found in *D. melanogaster* following successful (artificial) linear selection for high or low tooth number (Ahuja & Singh, 2008). After ten generations of relaxed selection, tooth number regressed back to intermediate, control numbers, demonstrating the action of net stabilizing selection on this component of the comb (Ahuja & Singh, 2008). Interestingly, males within the low tooth number lines were less likely to successfully mate if they had very few teeth compared to those that had more sex comb teeth. However, among the control and high tooth number lines, the effect of tooth number on mating success was nonsignificant (Ahuja & Singh, 2008).

Here, nonlinear post‐copulatory selection on the sex combs was stronger and disruptive when measured as sperm offence. As the significant eigenvalues (**λ**) are positive, it suggests that the fitness surface is concave and best described as a bowl (Fig. 3a; Hunt *et al*., 2009), and along the height of the bowl, fertilization success is approximately equivalent (Fig. 3b). Two other studies have shown that particular morphologies of *Drosophila* sex combs enhance competitive fertilization success. In *D. bipectinata*, artificial selection was used to develop lines with relatively short or long combs, and relatively long combs were found to confer an advantage during sperm offence (Polak & Simmons, 2009). In contrast, in a field study of *D. melanogaster*, sexual selection on the sex comb was disruptive (Robinson *et al*., 2012) as we report here.

The patterns of selection that we found may be explained if extreme combinations of sex comb components are most effective at grasping the female and aligning her genitalia during insemination, with intermediate combinations being less effective. Similar patterns of disruptive selection have been found in naturally selected traits (Brodie, 1992; Smith, 1993; Bolnick, 2004) and sexually selected traits (Blows *et al*., 2003), and in three of these studies, competition for limiting resources appears to generate this pattern of selection (Smith, 1993; Blows *et al*., 2003; Bolnick, 2004). For instance, the African finch exhibit small or large bill size and feed exclusively on soft‐ or hard‐seeded sedge, respectively (Smith, 1993). In the three‐spine stickleback, intraspecific competition selects for extreme trophic morphology (i.e. large or small gill raker length) (Bolnick, 2004), and female choice selects for rare male phenotypes in guppies (Blows *et al*., 2003). The wider implication of this pattern of selection is subject to debate but theoretically can force niche expansion (Roughgarden, 1972), sexual dimorphism (Slatkin, 1984; Bolnick & Doebeli, 2003) and speciation (Doebeli, 1996; Dieckmann & Doebeli, 1999).

### Nonlinear selection and genetic correlations between sex comb components

In this study, all genetic correlations between body size and the sex comb components were positive so that genotypes predisposing to larger size also result in longer combs with both more numerous and longer teeth. However, highest post‐copulatory, paternity offence (i.e. P2) was correlated with a long sex comb but few comb teeth (i.e. intermediate **m**_**1**_ and positive **m**_**2**_), yet the positive genetic covariance between these sex comb traits means that few male genotypes occupy this region of the landscape. This may reflect an underlying mechanistic constraint as sex combs are positively allometric (Sharma *et al*., 2011), so the scaling of sex comb trait components with body size largely prevents this combination. Evidence from other species suggests that the cause of genetic covariance may originate from developmental or functional constraints that place limits on trait combinations. For example, negative genetic covariance between the call rate and chirp duration of a cricket (Wagner *et al*., 2012) and ejaculate size and sperm quality in a cockroach (Moore *et al*., 2004) may reflect the energetics of calling (Wagner *et al*., 2012) and sperm production (Moore *et al*., 2004), whereas the negative covariance among colour pattern components in a guppy (Brooks & Endler, 2001) and the correlated evolution of beak morphology and vocal repertoire of Darwin's finches (Podos, 2001) may be due to physical constraints. For instance, in the guppy, spots occupied by one colour may preclude another (Brooks & Endler, 2001) and, in finches, beaks that become adapted for increased bite force are less able to perform rapid movements that are required for certain songs (Podos, 2001).

### Opportunity for sexual selection across selective episodes

*Drosophila simulans* belong to a clade in which female remating is infrequent and females can be more choosy after mating as they can use stored sperm to continue to produce offspring (Taylor *et al*., 2007, 2008a,b). More broadly in *Drosophila sp*, it is striking that secondary sexual traits, such as the sex comb, are only present in clades where females rarely remate. Theoretically, this should increase the variance in male mating success and thus the opportunity for selection on male secondary sexual characters (Emlen & Oring, 1977; Markow, 2002; Collet *et al*., 2012). Our results are partially consistent with this expectation, with no evidence of selection on male sex combs during any bout of selection when females are virgin, whereas we detect some nonlinear selection on sex combs when females are mated. For example, during precopulatory selection, virgin females rejected ~30% of male courtship displays and this increased to ~60% when females were mated which was matched by the detection of selection on the sex comb, albeit weak selection. Similarly, post‐copulatory sexual selection on sex combs was only significant for sperm offence – so selection on the sex combs is acting through males’ ability to displace a mated female's stored sperm.

## Conclusions

Given the lack of directional selection acting on the sex comb, formal analysis of the extent to which the covariance structure in **G** constrains a selection response is not particularly informative. Studies that have implemented this approach show that genetic covariances constrain evolution sometimes (Hine *et al*., 2004; Hunt *et al*., 2007a,b; Von Homrigh *et al*., 2007; Hall *et al*., 2010; Ingleby *et al*., 2014) but not always (Ingleby *et al*., 2014; Walling *et al*., 2014; Welch *et al*., 2014). Here, selection is nonlinear, comprising a combination of stabilizing and disruptive processes as described by the fitness surfaces. These forms of selection coupled with the lack of net linear selection may contribute to the maintenance of genetic variation and prevent male sex combs from evolving to a single optimal phenotype. Furthermore, data from other systems suggest that disruptive selection, as we find here, may be important for divergent evolution and speciation (Schluter, 2000).

## References

[jeb13015-bib-0001] Ahuja, A. & Singh, R.S. 2008 Variation and evolution of male sex combs in *Drosophila*: nature of selection response and theories of genetic variation for sexual traits. Genetics 179: 503–509. 18493067 10.1534/genetics.107.086363 PMC2390627

[jeb13015-bib-0002] Andersson, M. 1994 Sexual Selection. Princeton University Press, Princeton, New Jersey.

[jeb13015-bib-0003] Andersson, M.B. & Simmons, L.W. 2006 Sexual selection and mate choice. Trend Ecol. Evol. 21: 296–302. 10.1016/j.tree.2006.03.015 16769428

[jeb13015-bib-0533] Arnqvist, G. 1998 Comparative evidence for the evolution of genitalia by sexual selection. Nature 393: 784–786.

[jeb13015-bib-0004] Ashburner, M. , Golic, K.G. & Hawley, R.S. 2005 Drosophila: A Laboratory Handbook. Cold Spring Harbour Press, Cold Spring Harbour, NY.

[jeb13015-bib-0005] Bentsen, C.L. , Hunt, J. , Jennions, M.D. & Brooks, R. 2006 Complex multivariate sexual selection on male acoustic signalling in a wild population of *Teleogryllus commodus* . Am. Nat. 167: 102–116. 10.1086/501376 16670989

[jeb13015-bib-0006] Blows, M.W. & Brooks, R. 2003 Measuring nonlinear selection. Am. Nat. 162: 815–820. 14737718 10.1086/378905

[jeb13015-bib-0007] Blows, M.W. & Hoffmann, A.A. 2005 A reassessment of genetic limits to evolutionary change. Ecology 86: 1371–1384.

[jeb13015-bib-0008] Blows, M.W. , Brooks, R. & Kraft, P.G. 2003 Exploring complex fitness surfaces: multiple ornamentation and polymorphism in male guppies. Evolution 57: 1622–1630. 12940366 10.1111/j.0014-3820.2003.tb00369.x

[jeb13015-bib-0009] Bolnick, D.I. 2004 Can intraspecific competition drive disruptive selection? An experimental test in natural populations of sticklebacks. Evolution 58: 608–618. 15119444

[jeb13015-bib-0010] Bolnick, D.I. & Doebeli, M. 2003 Sexual dimorphism and adaptive speciation: two sides of the same ecological coin. Evolution 57: 2433–2449. 14686521 10.1111/j.0014-3820.2003.tb01489.x

[jeb13015-bib-0011] Brodie, E.D. III 1992 Correlational selection for colour pattern and antipredator behaviour in the garter snake *Thamnophis ordinoides* . Evolution 46: 1284–1298. 10.1111/j.1558-5646.1992.tb01124.x 28568995

[jeb13015-bib-0012] Brooks, R. & Endler, J.A. 2001 Direct and indirect sexual selection and quantitative genetics of male traits in guppies (*Poecilia reticulata*). Evolution 55: 1002–1015. 11430637 10.1554/0014-3820(2001)055[1002:daissa]2.0.co;2

[jeb13015-bib-0013] Chenoweth, S.F. & Blows, M.W. 2005 Contrasting mutual selection on homologous signal traits in *Drosophila serrata* . Am. Nat. 165: 281–289. 15729657 10.1086/427271

[jeb13015-bib-0014] Chenoweth, S.F. , Hunt, J. & Rundle, H. 2012 Analyzing and comparing the geometry of individual fitness surfaces In: The Adaptive Landscape in Evolutionary Biology (Svensson E. & Calsbeek R., eds), pp. 126–320. Oxford University Press, Oxford.

[jeb13015-bib-0015] Cheverud, J.M. 1984 Quantitative genetics and developmental constraints on evolution by selection. J. Theor. Biol. 110: 155–171. 6492829 10.1016/s0022-5193(84)80050-8

[jeb13015-bib-0016] Collet, J. , Richardson, D.S. , Worley, K. & Pizzari, T. 2012 Sexual selection and the differential effect of polyandry. Proc. Natl. Acad. Sci. USA 109: 8641–8645. 22592795 10.1073/pnas.1200219109 PMC3365207

[jeb13015-bib-0017] Coyne, J.A. & Beecham, E. 1987 Heritability of two morphological characters within and among natural populations of *Drosophila melanogaster* . Genetics 117: 727–737. 3123311 10.1093/genetics/117.4.727 PMC1203244

[jeb13015-bib-0018] Danielsson, I. 2001 Antagonistic pre‐ and post‐copulatory sexual selection on male body size in a water strider (*Gerris lacustris*). Proc. R. Soc. Lond. B 268: 77–81. 10.1098/rspb.2000.1332 PMC1087603 12123301

[jeb13015-bib-0019] Demary, K.C. & Lewis, S.M. 2007 Male courtship and paternity success in *Photinus greeni* fireflies. Evolution 61: 431–439. 17348952 10.1111/j.1558-5646.2007.00033.x

[jeb13015-bib-0020] Dieckmann, U. & Doebeli, M. 1999 On the origin of species by sympatric speciation. Nature 400: 354–357. 10432112 10.1038/22521

[jeb13015-bib-0021] Doebeli, M. 1996 A quantitative genetic competition model for sympatric speciation. J. Evol. Biol. 9: 893–909.

[jeb13015-bib-0022] Draper, N.R. & John, J.A. 1988 Response‐surface designs for quantitative and qualitative variables. Technometrics 30: 423–428.

[jeb13015-bib-0023] Eberhard, W.G. 1985 Sexual selection and Animal Genitalia. Harvard University Press, Cambridge, MA.

[jeb13015-bib-0024] Emlen, S.T. & Oring, L.W. 1977 Ecology, sexual selection and the evolution of mating systems. Science 197: 215–223. 327542 10.1126/science.327542

[jeb13015-bib-0025] Evans, J.P. , Zane, L. , Francescato, S. & Pilastro, A. 2003 Directional postcopulatory sexual selection revealed by artificial insemination. Nature 421: 360–363. 12540898 10.1038/nature01367

[jeb13015-bib-0026] Fear, K.K. & Price, T. 1998 The adaptive surface in ecology. Oikos 82: 440–448.

[jeb13015-bib-0027] Gilchrist, A.S. & Partridge, L. 1999 A comparison of the genetic basis of wing size divergence in three parallel body size clines of *Drosophila melanogaster* . Genetics 153: 1775–1787. 10581284 10.1093/genetics/153.4.1775 PMC1460863

[jeb13015-bib-0028] Gowaty, P.A. , Steinichen, R. & Anderson, W.W. 2002 Mutual interest between the sexes and reproductive success in *Drosophila pseudoobscura* . Evolution 56: 2537–2540. 12583593 10.1111/j.0014-3820.2002.tb00178.x

[jeb13015-bib-0029] Green, P.J. & Silverman, B.W. 1994 Nonparametric Regression and Generalized Linear Models. Glasgow: Chapman & Hall, Glasgow.

[jeb13015-bib-0030] Hall, M.D. , Lailvaux, S.P. , Blows, M.W. & Brooks, R.C. 2010 Sexual conflict and the maintenance of multivariate genetic variation. Evolution 64: 1697–1703. 20050914 10.1111/j.1558-5646.2009.00932.x

[jeb13015-bib-0031] Head, M.L. , Hunt, J. & Brooks, R. 2006 Genetic association between male attractiveness and female differential allocation. Ecol. Evol. 2: 341–344. 10.1098/rsbl.2006.0474 PMC1686179 17148398

[jeb13015-bib-0032] Hedge, S.N. & Krishna, M.S. 1997 Size‐assortative mating in *Drosophila malerkotliana* . Anim. Behav. 54: 419–426. 9268474 10.1006/anbe.1996.0485

[jeb13015-bib-0033] Hine, E. , Chenoweth, S.F. & Blows, M.W. 2004 Multivariate quantitative genetics and the lek paradox: genetic variance in male sexually selected traits of *Drosophila serrata* under field conditions. Evolution 58: 2754–2762. 15696753 10.1111/j.0014-3820.2004.tb01627.x

[jeb13015-bib-0034] Hosken, D.J. , Taylor, M.L. , Hoyle, K. , Higgins, S. & Wedell, N. 2008 Attractive males have greater success in sperm competition. Curr. Biol. 18: R553–R554. 18606122 10.1016/j.cub.2008.04.028

[jeb13015-bib-0035] Houle, D. 1992 Comparing evolvability and variability of quantitative traits. Genetics 130: 195–204. 1732160 10.1093/genetics/130.1.195 PMC1204793

[jeb13015-bib-0036] House, C.M. & Simmons, L.W. 2005 The evolution of male genitalia: patterns of genetic variation and covariation in the genital sclerites of the dung beetle *Onthophagus taurus* . J. Evol. Biol. 18: 1281–1292. 16135123 10.1111/j.1420-9101.2005.00926.x

[jeb13015-bib-0037] Hunt, J. , Wolf, J.B. & Moore, A.J. 2007a The biology of multivariate evolution. J. Evol. Biol. 20: 1–8. 17209992 10.1111/j.1420-9101.2006.01222.x

[jeb13015-bib-0038] Hunt, J. , Blows, M.W. , Zajitschek, F. , Jennions, M.D. & Brooks, R. 2007b Reconciling strong stabilizing selection with the maintenance of genetic variation in a natural population of black field crickets (*Teleogryllus commodus*). Genetics 177: 875–880. 17660544 10.1534/genetics.107.077057 PMC2034650

[jeb13015-bib-0039] Hunt, J. , Breuker, C.J. , Sadowski, J.A. & Moore, A.J. 2009 Male‐male competition, female mate choice and their interaction: determining total sexual selection. J. Evol. Biol. 22: 13–26. 19120810 10.1111/j.1420-9101.2008.01633.x

[jeb13015-bib-0040] Ingleby, F.C. , Hosken, D.J. , Flowers, K. , Hawkes, M.F. , Lane, S.M. , Rapkin, J. *et al* 2014 Environmental heterogeneity, multivariate sexual selection and genetic constraints on cuticular hydrocarbons in *Drosophila simulans* . J. Evol. Biol. 27: 700–713. 24779049 10.1111/jeb.12338

[jeb13015-bib-0041] Kingsolver, J.G. & Diamond, S.E. 2011 Phenotypic selection in natural populations: what limits directional selection? Am. Nat. 177: 346–357. 21460543 10.1086/658341

[jeb13015-bib-0042] Kopp, A. & True, J.R. 2002 Evolution of male sexual characters in the oriental *Drosophila melanogaster* species group. Evol. Dev. 4: 278–291. 12168620 10.1046/j.1525-142x.2002.02017.x

[jeb13015-bib-0043] Koref‐Santibanez, S. 2001 Effects of age and experience on mating activity in the sibling species *Drosophila pavani* and *Drosophila gaucha* . Behav. Genet. 31: 287–297. 11699601 10.1023/a:1012279325621

[jeb13015-bib-0044] Lande, R. & Arnold, S.J. 1983 The measurement of selection on correlated characters. Evolution 37: 1210–1226. 10.1111/j.1558-5646.1983.tb00236.x 28556011

[jeb13015-bib-0045] Lynch, M. & Walsh, B. 1998 Genetics and Analysis of Quantitative Traits. Sinauer Associates, Sunderland, MA.

[jeb13015-bib-0046] Manning, A. 1967 The control of sexual receptivity in female *Drosophila* . Anim. Behav. 15: 239–250. 6030948 10.1016/0003-3472(67)90006-1

[jeb13015-bib-0047] Markow, T.A. 2002 Female remating, operational sex ratio, and the arena of sexual selection in *Drosophila* species. Evolution 59: 1725–1734. 10.1111/j.0014-3820.2002.tb00186.x 12389717

[jeb13015-bib-0048] Markow, T.A. & Ricker, J.P. 1992 Male size, developmental stability, and mating success in natural populations of three *Drosophila* species. Heredity 69: 122–127. 1526852 10.1038/hdy.1992.104

[jeb13015-bib-0049] Markow, T.A. , Bustoz, D. & Pitnick, S. 1996 Sexual selection and a secondary sexual character in two *Drosophila* species. Anim. Behav. 52: 759–766.

[jeb13015-bib-0050] Mitchell‐Olds, T. & Shaw, R.G. 1987 Regression analysis of natural selection: statistical inference and biological interpretation. Evolution 41: 1149–1161. 10.1111/j.1558-5646.1987.tb02457.x 28563617

[jeb13015-bib-0051] Moore, P.J. , Harris, E. , Montrose, T. , Levin, D. & Moore, A.J. 2004 Constraints on evolution and postcopulatory sexual selection: trade‐offs among ejaculate characteristics. Evolution 58: 1773–1780. 15446429 10.1111/j.0014-3820.2004.tb00460.x

[jeb13015-bib-0052] Mousseau, T.A. & Roff, D.A. 1987 Natural selection and the heritability of fitness components. Heredity 59: 181–197. 3316130 10.1038/hdy.1987.113

[jeb13015-bib-0053] Okada, K. , Blount, J.D. , Sharma, M.D. , Snook, R.R. & Hosken, D.J. 2011 Male attractiveness, fertility and susceptibility to oxidative stress are influenced by inbreeding in *Drosophila simulans* . J. Evol. Biol. 24: 363–371. 21091568 10.1111/j.1420-9101.2010.02170.x

[jeb13015-bib-0054] Okada, K. , Katsuki, M. , Sharma, M. , House, C.M. & Hosken, D.J. 2014 Sexual conflict over mating in *Gnatocerus cornutus*? Females prefer lovers no fighters. Proc. R. Soc. B 281: 20140281. 10.1098/rspb.2014.0281 PMC4024292 24807253

[jeb13015-bib-0055] Parker, G.A. 1970 Sperm competition and its evolutionary consequences in the insects. Biol. Rev. 45: 525–567.

[jeb13015-bib-0056] Partridge, L. & Halliday, T. 1984 Mating patterns and mate choice In: Behavioural Ecology: An Evolutionary Approach (Krebs J.R. & Davies N.B., eds), pp. 222–250. Blackwell Scientific, Oxford.

[jeb13015-bib-0057] Phillips, P.C. & Arnold, S.J. 1989 Visualizing multivariate selection. Evolution 43: 1209–1222. 10.1111/j.1558-5646.1989.tb02569.x 28564514

[jeb13015-bib-0058] Pitcher, W. , Wolf, J.B. , Tregenza, T. , Hunt, J. & Dworkin, I. 2014 Evolutionary rates for multivariate traits: the role of selection and genetic variation. Phil. Trans. R. Soc. B 369: 20130252. 25002697 10.1098/rstb.2013.0252 PMC4084537

[jeb13015-bib-0059] Podos, J. 2001 Correlated evolution of morphology and vocal signal structure in Darwin's finches. Nature 409: 185–188. 11196640 10.1038/35051570

[jeb13015-bib-0060] Polak, M. & Simmons, L.W. 2009 Secondary sexual trait size reveals competitive fertilization success in *Drosophila bipectinata* Duda. Behav. Ecol. 20: 753–760.

[jeb13015-bib-0061] Polak, M. , Starmer, W.T. & Wolf, L.L. 2004 Sexual selection for size and symmetry in a diversifying secondary sexual character in *Drosophila bipectinata* Duda (Diptera: Drosophilidae). Evolution 58: 597–607. 15119443

[jeb13015-bib-0062] Pomiankowski, A. & Moller, A.P. 1995 A resolution of the lek paradox. Proc. R. Soc. B 260: 21–29.

[jeb13015-bib-0063] Promislow, D.E.L. , Smith, E.A. & Pearse, L. 1998 Adult fitness consequences of sexual selection in *Drosophila melanogaster* . Proc. Natl. Acad. Sci. USA 95: 10687–10692. 9724765 10.1073/pnas.95.18.10687 PMC27956

[jeb13015-bib-0064] Reynolds, R.J. , Childers, D.K. & Pajewski, N.M. 2010 The distribution and hypothesis testing of eigenvalues from the canonical analysis of the gamma matrix of quadratic and correlation selection gradients. Evolution 64: 1076–1085. 19863584 10.1111/j.1558-5646.2009.00874.x PMC2857515

[jeb13015-bib-0065] Robertson, F.W. 1957 Studies in quantitative inheritance XI. Genetic and environmental correlation between body size and egg production in *Drosophila melanogaster* . J. Genet. 55: 428–443. 10.1007/BF02715825 15240905

[jeb13015-bib-0066] Robinson, S.P. , Kennington, W.J. & Simmons, L.W. 2012 Assortative mating for relatedness in a large naturally occurring population of *Drosophila melanogaster* . J. Evol. Biol. 25: 716–725. 22321157 10.1111/j.1420-9101.2012.02466.x

[jeb13015-bib-0067] Roff, D.A. & Mousseau, T.A. 1987 Quantitative genetics and fitness: lessons from *Drosophila* . Heredity 58: 103–118. 3818341 10.1038/hdy.1987.15

[jeb13015-bib-0068] Rogers, D.W. , Denniff, M. , Chapman, T. , Fowler, K. & Pomiankowski, A. 2008 Male sexual ornament size is positively associated with reproductive morphology and enhanced fertility in the stalk‐eyed fly *Teleopsis* . BMC Evol. Biol. 8: 236. 18710553 10.1186/1471-2148-8-236 PMC2562384

[jeb13015-bib-0069] Rose, E. , Paczolt, K.A. & Jones, A.G. 2013 The contribution of premating and postmating selection episodes to total selection in sex‐role‐reversed gulf pipefish. Am. Nat. 182: 410–420. 23933729 10.1086/671233

[jeb13015-bib-0070] Roughgarden, J. 1972 Evolution of niche width. Am. Nat. 106: 683–718.

[jeb13015-bib-0071] Rowe, L. & Houle, D. 1996 The lek paradox and the capture of genetic variance by condition dependent traits. Proc. R. Soc. Lond. B 263: 1415–1421.

[jeb13015-bib-0072] Sakai, T. & Ishida, N. 2001 Circadian rhythms of female mating activity governed by clock genes in *Drosophila* . Proc. Natl. Acad. Sci. USA 98: 9221–9225. 11470898 10.1073/pnas.151443298 PMC55401

[jeb13015-bib-0073] Schluter, D. 2000 The Ecology of Adaptive Radiation. Oxford University Press, Oxford.

[jeb13015-bib-0074] Shackleton, M.A. , Jennions, M.D. & Hunt, J. 2005 Fighting success and attractiveness as predictors of male mating success in the black field cricket, *Teleogryllus commodus*: the effectiveness of no‐choice tests. Behav. Ecol. Sociobiol. 58: 1–8.

[jeb13015-bib-0075] Sharma, M.D. , Tregenza, T. & Hosken, D.J. 2010 Female mate preferences in *Drosophila simulans*: evolution and costs. J. Evol. Biol. 23: 1672–1679. 20546089 10.1111/j.1420-9101.2010.02033.x

[jeb13015-bib-0076] Sharma, M.D. , Tregenza, T. & Hosken, D.J. 2011 Sex combs, allometry, and asymmetry in *Drosophila* . Biol. J. Linn. Soc. 103: 913–934.

[jeb13015-bib-0077] Simmons, L.W. & Emlen, D.J. 2006 Evolutionary trade‐off between weapons and testes. Proc. Natl. Acad. Sci. USA 103: 16346–16351. 17053078 10.1073/pnas.0603474103 PMC1637585

[jeb13015-bib-0078] Slatkin, M. 1984 Ecological causes of sexual dimorphism. Evolution 38: 622–630. 10.1111/j.1558-5646.1984.tb00327.x 28555984

[jeb13015-bib-0079] Smith, T.B. 1993 Disruptive selection and the genetic basis of bill size polymorphism in the African finch *Pyrenestes* . Nature 363: 618–620.

[jeb13015-bib-0080] Snook, R.R. , Gidaszewski, N.A. , Chapman, T. & Simmons, L.W. 2013 Sexual selection and the evolution of secondary sexual traits: sex comb evolution in *Drosophila* . J. Evol. Biol. 26: 912–918. 23496332 10.1111/jeb.12105

[jeb13015-bib-0081] Spieth, H.T. 1974 Courtship behaviour in *Drosophila* . Annu. Rev. Entomol. 19: 385–405. 4205689 10.1146/annurev.en.19.010174.002125

[jeb13015-bib-0082] Stinchcombe, J.R. , Agrawal, A.F. , Hohenlohe, P.A. , Arnold, S.J. & Blows, M.W. 2008 Estimating non‐linear selection gradients using quadratic regression coefficients: double or nothing? Evolution 62: 2435–2440. 18616573 10.1111/j.1558-5646.2008.00449.x

[jeb13015-bib-0083] Taylor, P.D. & Williams, G.C. 1982 The lek paradox is not resolved. Theor. Popul. Biol. 22: 392–409.

[jeb13015-bib-0084] Taylor, M.L. , Wedell, N. & Hosken, D.J. 2007 The heritability of attractiveness. Curr. Biol. 17: R959–R960. 18029248 10.1016/j.cub.2007.09.054

[jeb13015-bib-0085] Taylor, M.L. , Wedell, N. & Hosken, D.J. 2008a Sexual selection and female fitness in *Drosophila simulans* . Behav. Ecol. Sociobiol. 62: 721–728.

[jeb13015-bib-0086] Taylor, M.L. , Wigmore, C. , Hodgson, D.J. , Wedell, N. & Hosken, D.J. 2008b Multiple mating increases female fitness in *Drosophila simulans* . Anim. Behav. 76: 963–970.

[jeb13015-bib-0087] Von Homrigh, A. , Higgie, M. , McGuigan, K. & Blows, M.W. 2007 The depletion of genetic variance by sexual selection. Curr. Biol. 17: 528–532. 17306541 10.1016/j.cub.2007.01.055

[jeb13015-bib-0088] Wagner, W.E. , Beckers, O.M. , Tolle, A.E. & Basolo, A. 2012 Tradeoffs limit the evolution of male traits that are attractive to females. Proc. R. Soc. B 279: 2899–2906. 10.1098/rspb.2012.0275 PMC3367788 22456890

[jeb13015-bib-0089] Walling, C.A. , Morrissey, M.B. , Foerster, K. , Clutton‐Brock, T.H. , Pemberton, J.M. & Kruuk, L.E. 2014 A multivariate analysis of genetic constraints to life history evolution in a wild population of red deer. Genetics 198: 1735–1749. 25278555 10.1534/genetics.114.164319 PMC4256783

[jeb13015-bib-0090] Walsh, B. & Blows, M.W. 2009 Abundant genetic variation + strong selection = Multivariate genetic constraints: a geometric view of adaptation. Annu. Rev. Ecol. Evol. Syst. 40: 41–59.

[jeb13015-bib-0091] Welch, A.M. , Smith, M.J. & Gerhardt, H.C. 2014 A multivariate analysis of genetic variation in the advertisement call of the gray treefrog, *Hyla versicolor* . Evolution 68: 1629–1639. 24621402 10.1111/evo.12397

[jeb13015-bib-0092] Wilson, A.J. , Réale, D. , Clements, M.N. , Morrissey, M.M. , Postma, E. , Walling, C.A. *et al* 2010 An ecologist's guide to the animal model. J. Anim. Ecol. 79: 13–26. 20409158 10.1111/j.1365-2656.2009.01639.x

[jeb13015-bib-0093] Wright, L.I. , Tregenza, T. & Hosken, D.J. 2009 Inbreeding, inbreeding depression and extinction. Conserv. Genet. 9: 833–843.

[jeb13015-bib-0094] Yenisetti, S.C. & Hedge, S.C. 2003 Size‐related mating and reproductive success in a drosophilid: *Phorticella striata* . Zool. Stud. 42: 203–210.

[jeb13015-bib-0095] Zera, A.J. & Harshman, L.G. 2001 The physiology of life history trade‐offs in animals. Annu. Rev. Ecol. Syst. 32: 95–126.

